# The effects of a regional telepathology project: a study protocol

**DOI:** 10.1186/1472-6963-12-64

**Published:** 2012-03-15

**Authors:** Marie-Claude Trudel, Guy Paré, Bernard Têtu, Claude Sicotte

**Affiliations:** 1 HEC Montréal, 3000, Côte-Ste-Catherine Road, Montréal, Canada, H3T 2A7; 2 Canada Research Chair in Information Technology in Health Care, HEC Montréal, Montréal, Canada; 3 Head of the Pathology Department, Centre Hospitalier Affilié Universitaire de Québec, Québec, Canada; 4 Faculty of Medicine, University of Montreal, Montréal, Canada

## Abstract

**Background:**

Telepathology, which is an emerging form of telemedicine in Canada, is defined as the electronic transmission of pathological images, usually derived from microscopes, from one location to another. There are various applications of telepathology, including case referral for an expert opinion, provision of an emergency service in the absence of a resident pathologist, and education. Until now, there has been relatively little use of telepathology for core diagnostic services in the absence of a local pathologist, but this practice is likely to increase in the future. The Laval University Integrated Health Network is in the process of deploying a telepathology system, primarily to provide an intraoperative frozen section service to small hospitals in sparsely populated areas which are experiencing a severe shortage of on-site pathologists. The telepathology project involves 17 hospitals located in five regions of eastern Quebec, Canada. This paper describes the study protocol that will be used to evaluate the benefits associated with the project.

**Methods/Design:**

A panel of experts was first assembled by Canada Health Infoway to agree on a set of benefits indicators that could be applied to all telepathology projects across Canada. Using the set of indicators as an input, we have developed a three-step study protocol. First, a survey questionnaire will be distributed to appraise the way pathologists, pathology technologists and surgeons perceive the telepathology system and its impacts. Second, a series of semi-structured interviews will be conducted with project leaders and telepathology users at sites that are representative of all the hospitals in the Laval University Integrated Health Network. The overall aim is to better understand the expected and unexpected effects of telepathology on health care professionals and patients as well as on the regional organization and delivery of care services. Finally, a pre-post design using secondary data is proposed to evaluate a wide array of tangible benefits to the patients, the health care providers, the hospitals, and the region as a whole.

**Discussion:**

The Laval University Integrated Health Network's telepathology project is expected to yield positive and significant results that are relevant internationally. Our findings will provide valuable information on the nature and extent of benefits associated with telepathology systems intended to provide an intraoperative frozen section service to remote hospitals experiencing a shortage of specialists.

## Background

Pathology is a scientific field concerned with the study of the nature and causes of diseases. Anatomic pathology, which is at the heart of this paper, is a medical specialty concerned with the diagnosis of diseases based on the gross, microscopic, chemical, immunologic, and molecular examination of organs, tissues, and whole bodies (autopsies). Because pathology examinations serve as the basis for a large percentage of decisions about diagnosis, treatment, hospital admission, and discharge, their annual number have increased in recent years in step with health care demand and, ultimately, with population growth and demographic aging. While an increase in the supply of pathologists would appear to be essential to maintaining patient care, the number of pathologists has declined in many developed countries over the last decade. This has been the case in Japan [1,2], Australia [3], the UK and New Zealand [4], as well as Canada [5], to name a few. In the province of Quebec - the site of our study - the supply of pathologists fell 10% from 1998 to 2008 [5]. Moreover, pathology is known to be unpopular among medical students [6], making it even more difficult to reverse this trend.

A shortage of pathologists has several consequences. The most direct is a longer turn-around time before a diagnosis can be made on a specimen [7]. Knowing that in the case of cancer - an area where pathology diagnoses are crucial - prompt diagnosis/treatment is particularly important, the repercussions of this labor shortage can be dramatic. The shortage also influences workload and quality assurance. When there are fewer people to do a job, workers become overloaded and it is difficult to guarantee good quality [6,8]. Finally, because of the shortage many pathologists are compelled to work past retirement age, and this may add to the overall quality problem. Once purely theoretical, such consequences were confirmed in recent years when news accounts about errors in anatomic pathology testing began to make national headlines. In addition to this shortage, pathology is gaining in complexity and pathologists working alone increasingly require support from colleagues. It is estimated that 10 to 20% of oncology cares require a second opinion [9].

One way to counteract the scarcity of pathologists and help them obtain a second opinion is to adopt telepathology, which is defined as the practice of pathology at a distance. Telepathology uses telecommunications technologies to facilitate the transfer of image-rich pathology data between distant locations for the purposes of diagnosis, education, and research [10]. Telepathology is performed in three modes: static, dynamic, and hybrid. When a pathologist is reviewing one or several preselected still images, he is working in static mode (also known as store-and-forward). In contrast, when a pathologist examines images while communicating live with the remote site where the specimen is located, he is working in dynamic mode. Finally, whenever a pathologist uses both methods to provide a diagnosis, he is working in hybrid mode [11].

Microscopic static examinations work well when there is no ambiguity as to the images the laboratory technologist should submit to the pathologist [12]. A microscopic dynamic examination involves the use of a robotized remote-controlled microscope by the pathologist to explore an entire slide surface, change the magnification level, or focus on and digitize a portion of interest. The glass slide is usually prepared by a well-trained laboratory technologist who must also position it within the microscope [13]. A macroscopic (or gross) dynamic examination is performed when the pathologist can first look at the whole specimen (e.g., tumour, organ, etc.) and talk to the technologist to orient the cuts. The histological section is then fixed on a glass slide and completely digitized by a slide scanner to provide a virtual slide of outstanding quality that can be remotely accessed and examined [14]. Virtual slides, as they are called, therefore constitute a newer form of microscopic static examination.

Once installed, a telepathology system can be used for distant primary diagnoses, expert referrals, quality assurance, and education [12]. For distant diagnoses in the absence of a local pathologist or a telepathology system, slides must be physically sent to another facility, hence delaying the diagnoses. With telepathology, the slides are examined remotely, either statically or dynamically, and diagnoses are swiftly provided. In some particular instances, however, pathology examinations are performed while the patient is still under anaesthetics and undergoing surgery, and the surgeon needs a pathology diagnosis to properly resume his procedure. For these specific examinations, called intraoperative frozen sections, delays are undesirable. When there is no local pathologist in a hospital and a surgical procedure requires an intraoperative frozen section examination, either the patient is transferred to another hospital, a visiting pathologist is called on site, or a procedure is performed in two steps (creating time for the slides to be sent and read elsewhere).

With telepathology, intraoperative frozen section analyses can be performed whenever a pathologist is available at a remote location. The procedure usually unfolds as follows: a laboratory technologist manipulates the specimen locally while it is filmed; a pathologist performs a gross examination of the specimen from another location examining the specimen and interacting with the technologist via a monitor and tells her where to cut; the technologist prepares one or more slides from the section then digitizes and stores them in a repository; and the pathologist accesses the images, performs his examination and provides his diagnosis directly to the surgeon by phone. The present study focuses specifically on this hybrid telepathology service: intraoperative frozen section examinations of virtual slides.

Examinations of intraoperative frozen sections have been performed via telepathology for over 20 years. In 1989, a Norwegian hospital decided to cover another facility located 400 km away using a motorized video-microscope that could be remotely controlled. The project was then expanded to four other hospitals [15]. In Japan, where experiments with telepathology began in 1982, intraoperative rapid diagnosis has been intensively used since the early 1990's to alleviate the effect of a severe shortage of pathologists - only 0.7% of the total number of physicians in Japan are pathologists [1]. In the United States, a fully implemented telepathology service was introduced between Veterans Affairs Medical Centers in Milwaukee (Wisconsin) and Iron Mountain (Michigan) in 1996. This service has been used, among other things, to examine intraoperative frozen section cases at Iron Mountain, which has had no on-site pathologist since that time [16]. In Canada, pathologists from the University Health Network (UHN) started using telepathology to examine frozen sections in 2004. More precisely, pathologists located at the Toronto General Hospital cover for Toronto Western Hospital and its affiliated Neuroscience Center.

We conducted a scoping review [17] to identify the nature and extent of telepathology research evidence. First, we searched two databases (PubMed and ABI Inform) with the keyword "telepathology" to obtain a general sense of the existing research on this topic. We chose these databases because we felt that they were offering a good sample of knowledge dissemination within the medical field (PubMed) as well as the business field (ABI Inform). Our queries, performed on September 9, 2011, resulted in 763 citations (730 found in PubMed, 25 found in ABI Inform and 8 found in both databases.) The titles and abstracts of these articles were skimmed and they appeared to be predominantly validations of the diagnostic accuracy of telepathology systems. At that point, we added the keyword "frozen section" to our search algorithm, as our study focuses on this type of examination. Our revised queries resulted in 72 citations (68 found in PubMed, three found in ABI Inform and one found in both databases). Four of these articles were excluded: three were not in English or French and one could not be found through our interlibrary system. Of the remaining 68 articles, 38 were concerned with diagnostic accuracy (19 of these articles also examined the time spent to arrive at these diagnoses), 10 were general papers introducing the topic/technology, nine were qualitative research on matters such as organizational consequences, learning, and cooperation in setups where telepathology was used (seven of them were subsections of a single research project by a single author), four were technical papers related to specific systems/features, and two were descriptive case studies presenting projects without evaluating them. Finally, only two articles were looking at the outcomes of telepathology, which is at the heart of our project. This literature dates back to 1991 when the first papers on the Norwegian installations were published.

According to Drummond et al. [18], evaluations must first be carried out on efficacy (can this theoretically work?), effectiveness (does this work in practice?), and availability (is this accessible to those who need it?) before an in-depth economic evaluation can be performed. The results of our scoping review confirm this evolution and suggest that the field is mature enough for more benefit assessments, economic or not. In this line of thought, the primary objective of this study is to evaluate the benefits of a major regional telepathology project implemented in 17 health care organizations located in eastern Quebec, Canada. The main goal of the project is to allow remote analysis of intraoperative frozen sections, although the telepathology system in place can also be used for other purposes, including secondary opinions and education sessions. As explained below, expected benefits encompass quality, access and productivity outcomes and will be measured at both the local and regional levels.

**Figure 1:**
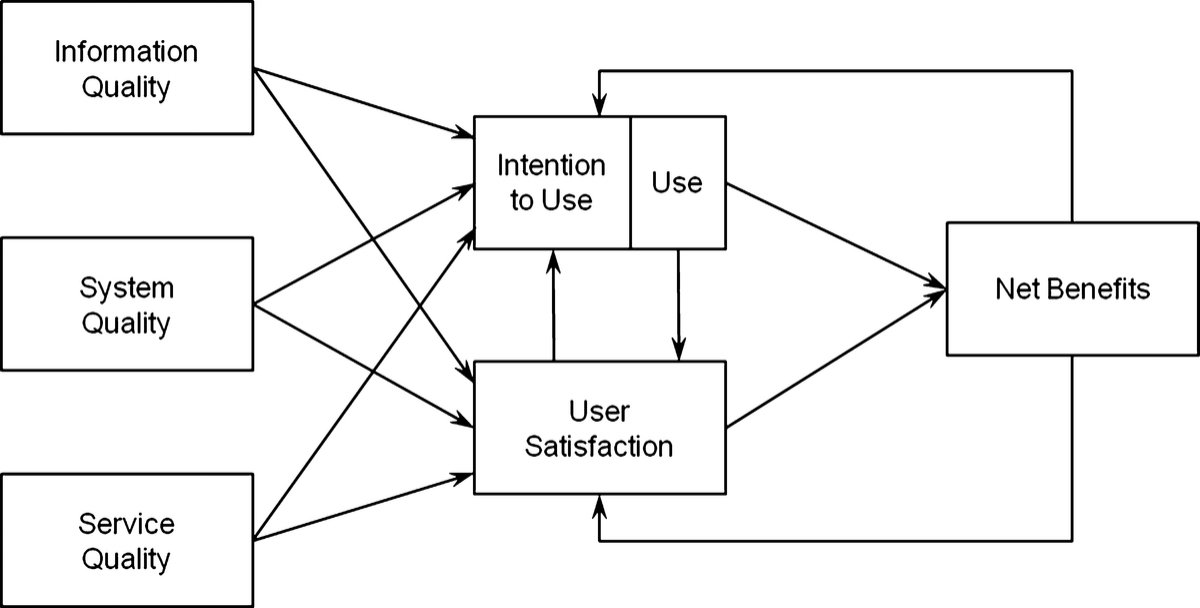
**Conceptual Framework (from DeLone and McLean, 2003)**.

Our evaluative approach is based on DeLone and McLean's information systems success model, illustrated in Figure 1. According to this framework, the individual benefits of an information system are derived from expressed satisfaction with the system and its use, which are in turn influenced by three factors: the perceived quality of the system, the perceived quality of the information contained in the system, and the perceived quality of the technical and organizational support offered. Finally, the greater the benefits at the individual level, the more the positive effects associated with use of the new system will be felt at the organizational and regional levels [19-21].

## Methods/Design

### Setting and project

The province of Quebec counts 7.9 million inhabitants and its health system is public. The health system is divided into four regions, each under the responsibility of a different University Integrated Health Network (UIHN). The mandate of each UIHN is to coordinate tertiary health care services, teaching and research provided by its faculty of medicine and its associated teaching hospitals so as to improve access to health care by streamlining relationships between primary care providers - doctors and regional hospitals - and upper-level care providers for specialized procedures. Each UIHN is also responsible for coordinating technology evaluation and health intervention methods in order to improve productivity and efficiency on its territory. In 2004, the Quebec Health Ministry asked each UIHN to submit a business case for a major regional telehealth project. The Laval University Integrated Health Network (hereinafter called the Laval UIHN) proposed a telepathology project, which is described next.

**Figure 2:**
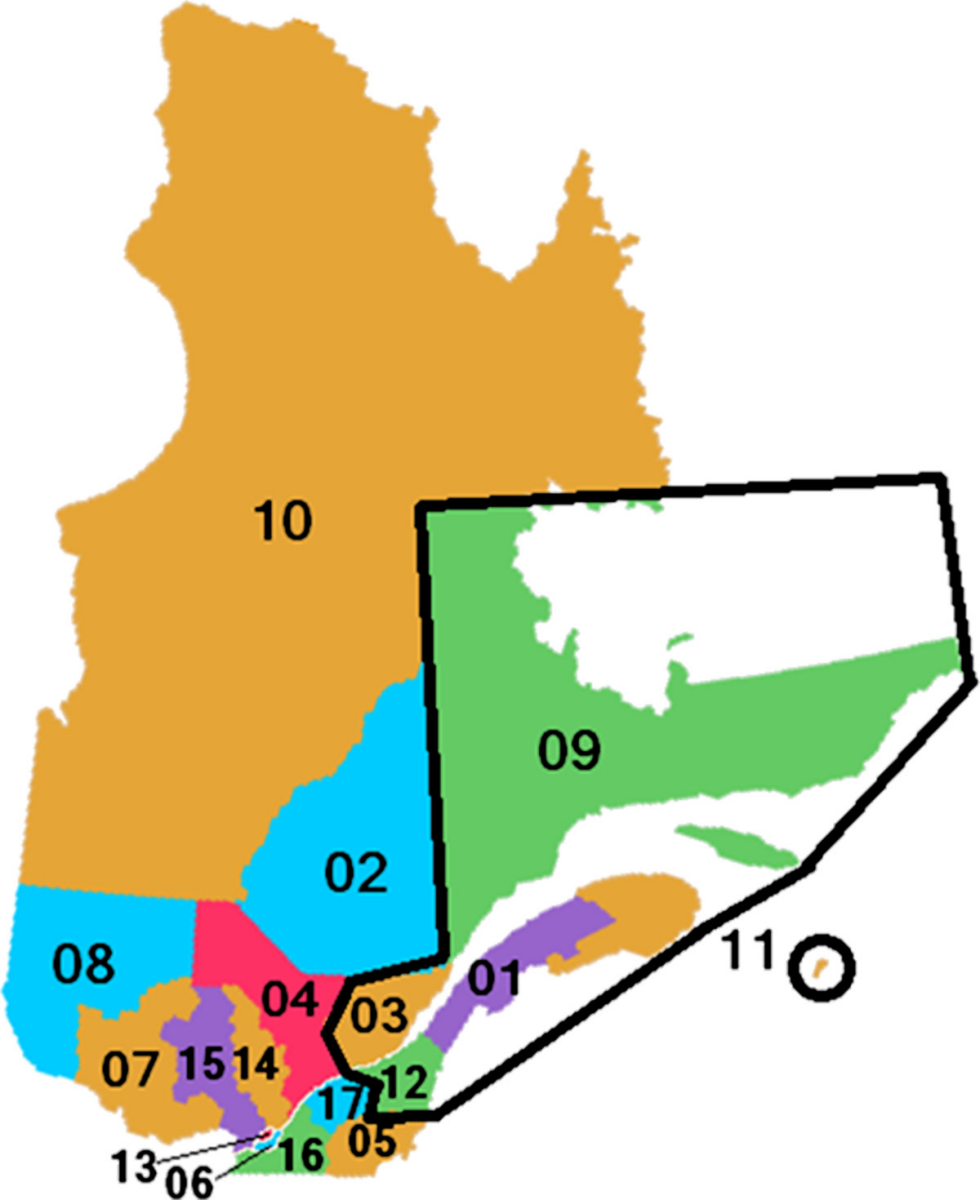
**Regions Underserved by the Laval UIHN in Eastern Quebec, Canada**.

The telepathology project involves 17 hospitals located in 5 geographic regions of eastern Quebec, Canada [22]. The area covered by the project accounts for approximately 19% of the province's total population and the territory spans 378,037 km^2 ^(approximately the size of Germany). Its population density is quite low. Figure 2 illustrates the province of Quebec and the area covered by the telepathology project, and Table 1 presents more information on the different regions.

**Table 1:** Profile of the Regions Underserved by the Laval UIHN

	Region 1	Region 3	Region 9	Region 11	Region 12
	**Bas-Saint-Laurent**	**Capitale Nationale**	**Côte-Nord**	**Gaspésie-Îles-de-la-Madeleine**	**Chaudière-Appalaches**

Type	Intermediary	University	Remote	Remote	Peripheral

Population	201,135	695,262	96,001	94,243	406,596

Area (km^2^)	22,405.07	19,285.71	300,281.83	20,937.69	15,128.14

Population density (per km^2^)	8.98	36.05	0.32	4.50	26.88

At the beginning of 2010, six of the 17 hospitals had no pathology laboratory and seven hospitals had a laboratory but were functioning with 0, 1 or 1.25 full-time equivalent pathologists (see Table 2). All 17 hospitals had surgeons who could execute procedures requiring intraoperative frozen section analyses. For these procedures to be performed where there was no on-site pathologist, four courses of actions were possible: (1) a visiting pathologist could be appointed, (2) the patient could be transferred to another hospital, (3) the operation could be completed in two stages (the specimen would be transported and analyzed elsewhere and the patient would be reopened if the diagnosis was positive), or (4) the surgery could be performed more aggressively (i.e. by removing more of the organ in order to "play it safe").

**Table 2:** Profile of the Sites Involved in the Laval UIHN's Telepathology Project

Region	Type	Location of the site	Distance from CHUQ's central laboratory	Presence of a pathology laboratory	Number of pathologists on site	Status in May 2011
1 Bas-Saint-Laurent	Intermediary	Rimouski	313 km	Yes	2	Equipment installed and training completed
		
		Rivière-du-Loup	206 km	Yes	1.25	Equipment installed and training completed

3 Capitale Nationale	University	Quebec City CHUQ - HD	0	Yes	15*	Equipment installed and training completed
				
		Quebec City CHUQ - CHUL	8.4 km	Yes		Equipment installed and training completed
				
		Quebec City CHUQ - HSFA	3.3 km	Yes		Equipment installed and training completed
		
		Quebec City CHAUQ - HSS	3.3 km	Yes	12*	Equipment installed and training completed
				
		Quebec City CHAUQ - HEJ	3.6 km	Yes		Equipment installed and training completed
		
		Quebec City Hôpital Laval	8.1 km	Yes	3	Equipment installed and training completed
		
		La Malbaie*	145 km	No	0	Equipment installed and training completed
		
		Baie-Saint-Paul*	93 km	No	0	Delivered

9 Côte-Nord	Remote	Sept-Îles	672 km	Yes	0	Equipment installed and training completed
		
		Baie-Comeau	444 km	Yes	1	Equipment installed and training completed

11 Gaspésie-Îles-de-la-Madeleine	Remote	Gaspé	687 km	Yes	1	Equipment installed and training completed
		
		Chandler	720 km	No	0	Equipment installed
		
		Saint-Anne-des-Monts	494 km	No	0	Equipment installed
		
		Les-Îles-de-la-Madeleine	1,153 km	No	0	Equipment installed
		
		Maria	587 km	No	0	Equipment installed

12 Chaudière-Appalaches	Peripheral	Lévis	31.3 km	Yes	3	Equipment installed and training completed
		
		Saint-Georges	107 km	Yes	1	Equipment installed and training completed
		
		Thedford Mines	111 km	Yes	0	Equipment installed and training completed
		
		Montmagny	80 km	Yes	1	Equipment installed and training completed

* Numbers provided for the merged organization

** These two sites account for one hospital (CH Charlevoix)

In 2006, the Laval UIHN decided to invest in a large telepathology project to offer the population in peripheral, intermediary and remote regions improved access and continuity with respect to specialized services. The CAN$6.2 million project is financed by the Quebec Health Ministry and Canada Health Infoway, an independent not-for-profit corporation that works as a strategic investor of funds provided by the Canadian government. In May 2011, the status of the project was as follows: the equipment had been delivered, physically installed and configured, and biomedical technicians, laboratory technologists and pathologists had been trained at 14 of the 21 sites. The equipment had been delivered, physically installed and configured but training had not been given at five other sites, mainly the ones that had to build a pathology laboratory before receiving the equipment. Finally, at the last site, the equipment had been delivered but further steps were delayed because the building posed a security risk, according to a recent seismic study. The equipment delivered to each site comprised a slide scanner (Nanozoomer, *Hamamatsu Photonics, Shizuoka Prefecture, Japan*), a macroscopic imaging station (Pathstand 40, *Diagnostic Instruments, Sterling Height, USA*), visualization and communication software (mScope, *Aurora Interactive Ltd., Montreal, Canada*), computer stations, and storage servers.

As mentioned above, the main applications for telepathology are distant primary diagnosis, expert referrals, and education [12]. The Laval UIHN's priority in this project is to have the telepathology system used for intraoperative examinations and, to a lesser extent, expert referrals, although utilization for other purposes (quality assessment, education, etc.) is not forbidden. Lastly, an important feature of the Laval UIHN project is the absence of a core laboratory to provide telepathology services to each facility involved. Instead, each hospital had to enter into a partnership agreement with one or more facilities of their choice and test their configurations (hardware, software and telecommunication links.) Ultimately, each hospital should be linked to every other hospital, which would make a rotating 24-hour schedule feasible.

### Research design

This study uses a mixed method design that integrates case studies, survey questionnaires and a quantitative pre-post evaluation. As a first step, a panel of experts was assembled by the Benefits Realization and Quality Improvement department at Canada Health Infoway. This panel consisted of 26 people engaged in various telepathology projects across Canada. Panel members either were pathologists, laboratory technologists, or independent academic researchers. The goal of this panel was to agree on a set of indicators that could be applied to most Canadian telepathology projects. The panel met via teleconference once a month for 4 months in order to share opinions. As mentioned before, the theoretical framework driving the effort was DeLone and McLean's information systems success model [19,21]. The final list of indicators, shown in Table 3, was approved in May 2011.

**Table 3:** List of Indicators and Measures

Construct		
**Dimension**		**Question**

SYSTEM QUALITY		

Ease of use		Generally speaking, the telepathology system is easy to use.
		
		It is easy to master the telepathology system functionalities.
		
		The graphical interfaces of the telepathology system are clear and easy to understand.
		
		It is easy to find slides from the other facilities in the telepathology system.
		
		It is easy to send slides and information to other facilities using telepathology.

Screen quality		The PC screens available in our facility are of acceptable quality.
		
		The screens dedicated to the telepathology system are of acceptable quality.
		
		The quality of the screens encourages me to use the telepathology system.

Systems integration		The telepathology system and the laboratory information system (LIS) are well integrated.
		
		The telepathology system, the LIS and the dictation subsystem are well integrated.
		
		Joint use of the telepathology system/LIS/dictation subsystem makes the work easier.
		
		When the data on a patient come from different facilities, the telepathology/LIS systems provide well-integrated information.
		
		When the data on a patient come from different facilities, the telepathology system/LIS/dictation subsystem provide well-integrated information.

Reliability		The telepathology system is rarely offline because of technology breakdowns.
		
		Unexpected service outages of the telepathology system rarely occur.
		
		Use of the telepathology system is uninterrupted because it is bug-free.

Accessibility		We have a sufficient number of telepathology work stations.
		
		I have rarely had to wait for access to a work station in order to use the telepathology system.
		
		I have easy access to the telepathology system from my home computer.

Response time		I have the impression that slides are digitized quickly.
		
		I have the impression that the macroscopic images are fluid.
		
		I have the impression that the microscopic images download quickly.
		
		We have quick access to images from other facilities.
		
		Setting aside image acquisition, the telepathology system responds quickly.

Perceived Usefulness		Overall, the telepathology system provides a complete range of functionalities that support my work as a professional.
		
		My clinical practices are very well harmonized with the telepathology system.
		
		Using the telepathology system is compatible with all aspects of my tasks.

INFORMATION QUALITY		

Data quality		The slide images produced locally in my facility are: • Complete • Reliable and precise • Well organized and carefully presented • Available in a timely manner • Secure and confidential
		
		The slide images produced externally in other facilities are: • Complete • Reliable and precise • Well organized and carefully presented • Available in a timely manner • Secure and confidential

SERVICE QUALITY		

Technical support		The pilots or super-users in my hospital who provide technical assistance for the telepathology system: • Are easy to reach • Provide quick service • Are competent • Pay attention to user needs • Are able to find satisfactory solutions
		
		The staff from the regional support centre who provide technical assistance for the telepathology system: • Are easy to reach • Provide quick service • Are competent • Pay attention to user needs • Are able to find satisfactory solutions

Training		I am satisfied with the training I received on using the telepathology system.
		
		I learned how to use the telepathology system properly because of the training I received.
		
		I apply what I have learned during my training on the telepathology system.
		
		I can better use the telepathology system because of the training I received.

USER SATISFACTION		

		Overall, my experience using the telepathology system has been satisfactory.
		
		I enjoy using the telepathology system in my work.
		
		Overall, examining digitized slides on a monitor is more satisfying than examining glass slides with a microscope.
		
		Being able to obtain a second opinion via the telepathology system satisfies me greatly.
		
		Being able to perform intraoperative frozen section analyses via telepathology satisfies me greatly.
		
		Being able to obtain an intraoperative rapid diagnosis via telepathology satisfies me greatly.
		
		Being able to use the telepathology system for immunohistochemistry cases satisfies me greatly.

USE		

Breadth		I use a wide range of the telepathology system's functionalities.

Type		How often do you...
		
		...use the telepathology system to fulfill your functions?
		
		...use the telepathology system to get/provide a second opinion?
		
		...use the telepathology system to get/provide a intraoperative frozen section diagnosis?
		
		...use the telepathology system for immunochemistry cases?

BENEFITS		

Individual impacts		Using the telepathology system improves the quality of my diagnoses.
		
		The telepathology system improves my confidence level when I perform some surgical procedures.
		
		Some of my surgical procedures are less invasive because of the telepathology system.
		
		Using the telepathology system saves me some of the time I used to spend moving about.
		
		The telepathology system reduces my sense of professional isolation.

Organizational impacts	Quality	The telepathology system's tracking mode reduces the risk of medical errors.
		
		The quality of patient care is improved because of the telepathology system.
	
	Efficiency	The telepathology system reduces the time between a surgical procedure and the availability of a preliminary report.
		
		The telepathology system reduces the time between the moment a slide is available for reading and the final diagnosis.
	
	Effectiveness	The telepathology system reduces the number of surgical procedures postponed due to a lack of pathologists.
		
		The telepathology system reduces the number of surgical procedures performed in two stages.
	
	Retention/ Recruitment	The telepathology system helps retain workers from different pathology professions (pathologists, pathology technologists, etc.) in our facility.
		
		The telepathology system helps recruit workers in different pathology professions for our region.
		
		The telepathology system makes the various pathology professions more attractive.

Regional impacts	Accessibility	The telepathology system has improved the accessibility of pathology services.
		
		The pathology coverage rate is improved because of telepathology.
		
		Fewer patients are transferred to regional or tertiary sites because of telepathology.
	
	Continuity	Patients enjoy improved continuity of care because of telepathology.

Our evaluation will be conducted in two separate phases. Phase 1 will focus on individual perceptions and will be divided into two parts. First, a survey questionnaire has been developed to appraise how users perceive the telepathology system and its impacts. The questionnaire was adapted from a recent digital medical imaging evaluation study that used the same framework as a conceptual foundation [23]. Questions were reformulated to reflect the different systems, work processes and settings and confirm the instrument's validity. Three versions of the questionnaire instrument are now ready to be pre-tested with a small group of pathologists (version 1), pathology technologists (version 2) and surgeons (version 3) who have been involved in telepathology projects elsewhere in Canada. Following the pre-test, a final version of each instrument will be prepared and gradually administered to all the pathologists (N = 48), pathology technologists (N = 60), and surgeons (N = 30) involved in the Laval UIHN project. Questionnaires will be distributed approximately 6 months after the "go live" at each site.

As a second step, a series of semi-structured interviews will be conducted concurrently with both project leaders and telepathology users at sites representative of all types of establishments - remote, intermediary, and teaching hospitals. The overall goal is to better understand the effects of telepathology on health care professionals, patients, and the overall organization and delivery of care services in different types of organizations. The data collection process will continue until theoretical saturation [24] is reached; i.e. when additional qualitative data no longer contributes anything new about the constructs in our conceptual framework and the relationships among them. Interview guides containing the specific issues to be discussed with the pathologists, pathology technologists, and surgeons were developed and will be used at each interview. Table 4 synthesizes the key constructs to be investigated with each group of respondents in Phase 1.

**Table 4:** Constructs and Categories of Respondents

Constructs	Pathologists	Technologists	Surgeons
Information quality	X		

System quality	X	X	

Service quality	X	X	

Use	X	X	

Intention to use	X	X	

User satisfaction	X	X	X

Net benefits	X	X	X

While Phase 1 is concerned with users' perceptions, Phase 2 will allow us to evaluate objective or factual benefits to the hospitals (e.g., a decrease in the number of cancelled surgical procedures due to pathology-related issues), the patients (e.g., a decrease in the average time between a medical diagnosis and a first surgical intervention) and the UIHN as a whole (e.g., a decrease in the number of patients transferred to a regional or tertiary hospital for surgery). To achieve this, pre-post analyses will be performed using data from different information systems - i.e. laboratory information systems, operating room information systems, and telepathology visualization and communication systems - available at each hospital. More specifically, data from these systems will be obtained for two 6-month periods: post implementation data will be obtained for the six first months after system deployment, and pre implementation data will be gathered for the corresponding 6 months of the previous year.

Given the exploratory nature of our study, it was decided to limit our sample to two specific procedures: sentinel lymph node analyses for breast cancer and surgical margin analyses for colorectal cancer. According to the Laval UIHN authorities, these procedures are among the most common ones requiring intraoperative frozen section diagnoses. In order to capture a broad range of effects, both anticipated and unanticipated, and to better understand the nature of the effects according to the context, our pre-post analysis will be conducted in three different settings. First, we will investigate a remote hospital where telepathology was deployed in order to avoid a break in services; the on-site pathologist left between the pre and post periods (thereby leaving the site with no pathologist). Our second setting is a remote hospital where pathology services were nonexistent. With the arrival of telepathology, a mini-laboratory was installed at this site, and a pathology technologist was trained so that medical interventions requiring pathology services could now be provided. Finally, we will investigate the effects of telepathology at a regional hospital where a number of pathologists left between the pre and post periods without being replaced, causing breaks in services.

### Data analysis

The psychometric properties of our survey measures will be assessed first. For one thing, the reliability and construct validity of all scales will be measured. One interpretation of the reliability criterion is the internal consistency of a test, i.e. the extent to which the items are homogeneous [25]. In this sense, reliability refers to the accuracy or precision of a measuring instrument. This will be tested by calculating a Cronbach's alpha for each construct. Convergent validity refers to whether the items comprising a scale behave as if they are measuring a common underlying construct. Hence, in order to demonstrate convergent validity, items that measure the same construct (i.e. a trait) should be highly correlated with one another [25]. Discriminant validity is concerned with the ability of a measurement item to differentiate between the concepts being measured. We will thus compare the square root of the variance shared by the constructs and their measures to the correlations between the constructs. Following this assessment of the measurement model, descriptive statistics will be computed and the associations presented in Figure 1 will be tested using linear stepwise regression analyses. Statistical tests will also be performed to determine whether significant differences can be found by type of institution (local, regional, university) and type of health care professional (pathologists, technologists, surgeons.) Given our small sample size, non-parametric tests such as Kruskal-Wallis and Mann-Whitney will be performed. The SPSS statistical package will be used to run these analyses at a 5% significance level.

As for the qualitative data, interview transcripts and observation notes will be analyzed using the NVivo software package. The constructs in our conceptual framework (Figure 1) will be critical to developing our coding scheme and interpreting the qualitative material [26]. Open coding will also be performed so that any unforeseen themes that might emerge from the data will also be captured [27]. It is worth mentioning that, in order to increase reliability, all interviews will be coded by two researchers.

Lastly, a paired two-sample test of means (t-test) will be conducted to determine whether any differences found between pre and post measures are significant (based on the list of indicators in Table 5). Given that two common procedures were selected for the pre-post analyses, we expect our sample size to be large enough to allow the use of a parametric test.

**Table 5:** Key Indicators for the Pre-Post Analyses

Usage types	Indicators
Second opinions	Number of second opinions sought
	
	Average delay between the request for a second opinion and its reception

Intraoperative frozen section analyses	Number of intraoperative diagnoses performed/number of surgical procedures performed
	
	Number of surgical procedures performed at the local or regional site
	
	Number of cancelled surgical procedures due to pathology related issues
	
	Number of surgical procedures performed in two stages
	
	Number of patients transferred to a regional or a tertiary hospital for surgery
	
	Coverage rate of in situ pathologists
	
	Coverage rate of visiting pathologists
	
	Average time between a medical diagnosis and a first surgical procedure
	
	Average time between a surgical procedure and a preliminary pathology report
	
	Average time between the moment the slide is ready to be read and the final pathology diagnosis is provided

### Ethics considerations

This research project has been assessed with regard to the ethical conduct for research involving humans and meets the requirement of HEC Montréal's Research Ethics Board. A notice has been issued to this effect on December 14, 2011.

## Discussion

In the last 30 years, the number of telemedicine applications has multiplied due to advancements in telecommunications. One of these applications, telepathology, has fostered considerable academic research since the mid-1980s. The extant literature has mainly followed the path described by Drummond et al. [18], and evaluations have so far been carried out on reliability, efficacy, effectiveness, and availability [e.g., [12,28]]. For frozen section examinations, the accuracy of the technique has been demonstrated [e.g., [29,30]] as has its speed [e.g., [14,31]]. However, very few empirical studies have evaluated the benefits of telepathology at the patient, provider, organizational and regional levels.

Using a mixed-method design that combines qualitative and quantitative data collection methods, the present study attempts to fill this gap. More specifically, our research will add to the extant body of knowledge by offering a thorough evaluation of a large telepathology project, assessing outcomes at multiple levels of analysis. To our knowledge, our study will be the first to examine a regional telepathology project by focusing on intraoperative frozen section analyses. Our study will also be the first to perform an exhaustive pre-post evaluation using secondary data from operating room and other health information systems. By drawing a clearer picture of the benefits to be obtained by deploying a large regional telepathology project, our study should help decision makers and regional authorities redistribute regional workforces around optimal workflows that take advantage of telepathology. Ultimately, positive results should influence health policy decisions targeted at broader societal and environmental issues.

Notwithstanding these anticipated contributions, our study protocol has limitations and, hence, more research will be needed to further explore the implications of our work. For one thing, we must acknowledge the usual limitations on generalizability associated with collecting data through mail questionnaires as well as the cross-sectional nature of Phase 1. Significantly, even though we will be able to compare contrasting situations by investigating a wide variety of settings in Phase 2, the fact remains that this research is limited to the study of a single telepathology project and our results will be context-dependent. Indeed, the province of Quebec has its own specificities in terms of the healthcare system (public), density of population (medium to very low), physicians' remuneration mode (fee-for-service) and pathologist shortage (moderately severe). Therefore, more evaluative studies will need to be conducted in other regions or countries to enhance the generalizability of our findings.

To our knowledge the ongoing telepathology project at the Laval UIHN is unique in Canada and is expected to yield significant results that are relevant both nationally and internationally. Results will provide valuable information to healthcare managers and decision makers who must find more effective and efficient ways to provide accessible care services to patients and, more specifically, patients living in rural regions. A demonstration of both perceived and actual impacts will likely contribute to the promotion of telepathology at the individual (patient and provider), organizational and regional levels. We hope that the study protocol provided here will guide other researchers interested in evaluating such projects and contribute to the overall development of a solid knowledge base regarding the range of benefits associated with telepathology in health care organizations.

## Abbreviations

UHN: University health network; UIHN: University integrated health network.

## Competing interests

The authors declare that they have no competing interests.

## Authors' contributions

MCT, GP and CS led the design of the study. MCT and GP developed the instruments and drafted the manuscript. BT and CS participated in the critical review of the manuscript. All authors gave final approvals on the version of the manuscript submitted for publication.

## Pre-publication history

The pre-publication history for this paper can be accessed here:

http://www.biomedcentral.com/1472-6963/12/64/prepub
